# Ketogenic diets in healthy dogs induce gut and serum metabolome changes suggestive of anti‐tumourigenic effects: A model for human ketotherapy trials

**DOI:** 10.1002/ctm2.1047

**Published:** 2022-09-23

**Authors:** Karin Allenspach, Dana C. Borcherding, Chelsea A. Iennarella‐Servantez, Allison P. Mosichuk, Todd Atherly, Dipak Kumar Sahoo, Aarti Kathrani, Jan S. Suchodolski, Agnes Bourgois‐Mochel, Mariana Rossoni Serao, Nick V. Serao, Auriel Willette, Beatriz Agulla Perez, Vojtech Gabriel, Sichao Mao, Logan Kilburn, Viet Dang, David Borts, Luciana L. Almada, Martin E. Fernandez‐Zapico, Gregory J. Phillips, Albert E. Jergens, Jonathan P. Mochel

**Affiliations:** ^1^ Departments of Veterinary Clinical Sciences Iowa State University College of Veterinary Medicine Ames Iowa USA; ^2^ Department of Biomedical Sciences Iowa State University College of Veterinary Medicine Ames Iowa USA; ^3^ Royal Veterinary College University of London London UK; ^4^ Gastrointestinal Laboratory Texas A&M University College of Veterinary Medicine & Biomedical Sciences College Station Texas USA; ^5^ Department of Animal Science Iowa State University Ames Iowa USA; ^6^ Food Science and Human Nutrition Iowa State University Ames Iowa USA; ^7^ Veterinary Diagnostics Laboratory Iowa State University Ames Iowa USA; ^8^ Schulze Center for Novel Therapeutics, Division of Oncology Research Mayo Clinic Rochester Minnesota USA; ^9^ Department of Veterinary Microbiology and Preventive Medicine Iowa State University Ames Iowa USA

1

Dear Editors,

In this study, we show that ketogenic diets (KD) in healthy dogs produce significant shifts in the serum metabolome which collectively suggest down‐regulation of the glycolytic and amino‐acid‐dependent metabolism of many tumour types. As dogs represent valuable models for preclinical evaluation of oncologic treatment modalities in humans,^1^ the data presented in this study will provide a resource upon which future investigations on the mechanistic effect of KDs on various tumours can be based.

KDs are defined as diets high in fat, low in carbohydrate and a protein content to meet requirements. Several cancers in humans have anecdotally been described to respond to KDs and have well‐characterized clinical analogues in dogs. However, there is a paucity of evidence‐based clinical studies available on the effect of KD in clinical patients. Dogs have adapted their physiology and metabolism to digest a similar diet to that of humans through domestication.^2^ Consequently, the composition of the dog microbiome is taxonomically and functionally much more similar to that of humans than that of mice.^3^ This is important as the clinical effects of KDs are thought to be mediated in part by modulation of the intestinal microbiome. We therefore hypothesized that using KD *in the dog model* would allow for an improved mechanistic analysis of sequential events on the serum metabolome as compared to mouse models. Eventually, clinical anti‐tumour effects of dietary interventions such as KD could be evaluated in clinical trial of dogs diagnosed with cancers, giving access to more informed pre‐clinical translational data before clinical trials in humans.

For the present study, two KDs (KD1 and KD2) were fed for 2 weeks each to eight healthy adult Beagle dogs after feeding a baseline diet (BSLN) (Figure 1A, Tables S1A, S1B and S2A, S2B).

FIGURE 1Short‐term ketogenic diet (KD) increases mucosal abundance of select bacteria in healthy dogs. (A) Schematic diagram of study design and sample collection in eight healthy beagle dogs. Dogs were fed baseline control diet (BSLN; 28.9 g/1000‐kcal ME), then high‐fat‐low carbohydrate diets 1 or 2 (KD1: 64.1, KD2: 79.3 g/1000‐kcal ME) or vice versa for 2 weeks each in randomized order. Endoscopic biopsy tissue, fecal and serum samples were collected after BSLN, KD1 and KD2. Dry matter/weight analysis of KD1 and KD2 as well as discriminant analyses of the diets are described in Tables S1 and S2, respectively. (B) Analysis of the fecal microbiome, conducted by shallow shotgun sequencing, showed distinct clustering and separation between BSLN diet and KD2 by Bray–Curtis principal coordinate analysis (PCoA) of operational taxonomic units (OTU). (C) The abundance of *Lactobacillales* and *Erysipelotrichales* decreased, whereas *Fusobacteriales*, *Bifidobacteriales*, *Selenomonadales*, *Aeromonadales* and *Enterobacteriales* increased with short‐term KD2 versus BSLN diet (*p* < .05). (D and E) Mucosal‐adherent *Eubacterium rectale/Clostridium coccoides* cluster (Erec) bacteria were increased after 2 weeks of KD2 (46% fat) versus KD1 (32% fat) in healthy dogs, as determined by fluorescent in situ hybridization (FISH) in colon biopsies of eight healthy beagle dogs. (D) Representative images (left) of Erec bacteria (orange) and total bacteria (EUB, green) in dog colon tissue after KD. (E) Boxplot graph of FISH analysis of Erec bacteria counts after KD1 versus KD2. *p* < .05. Scale bar = 100 μm. (F and G) Short‐term feeding of KD significantly alters faecal metabolites in healthy dogs. (F) Random forest analysis of untargeted metabolomics of canine fecal samples on BSLN and after 2 weeks of KD2 feeding. Cholesterol was one of the most significantly altered metabolites in the fecal samples of dogs after being fed a short‐term KD. (G) Boxplots of identifiable fecal metabolites that differed between the diets: Cholesterol and tocopherol acetate were significantly decreased after KD2, whereas beta‐sitosterol, zymosterol, gamma‐tocopherol and maltitol were increased after KD2.

**Figure:**
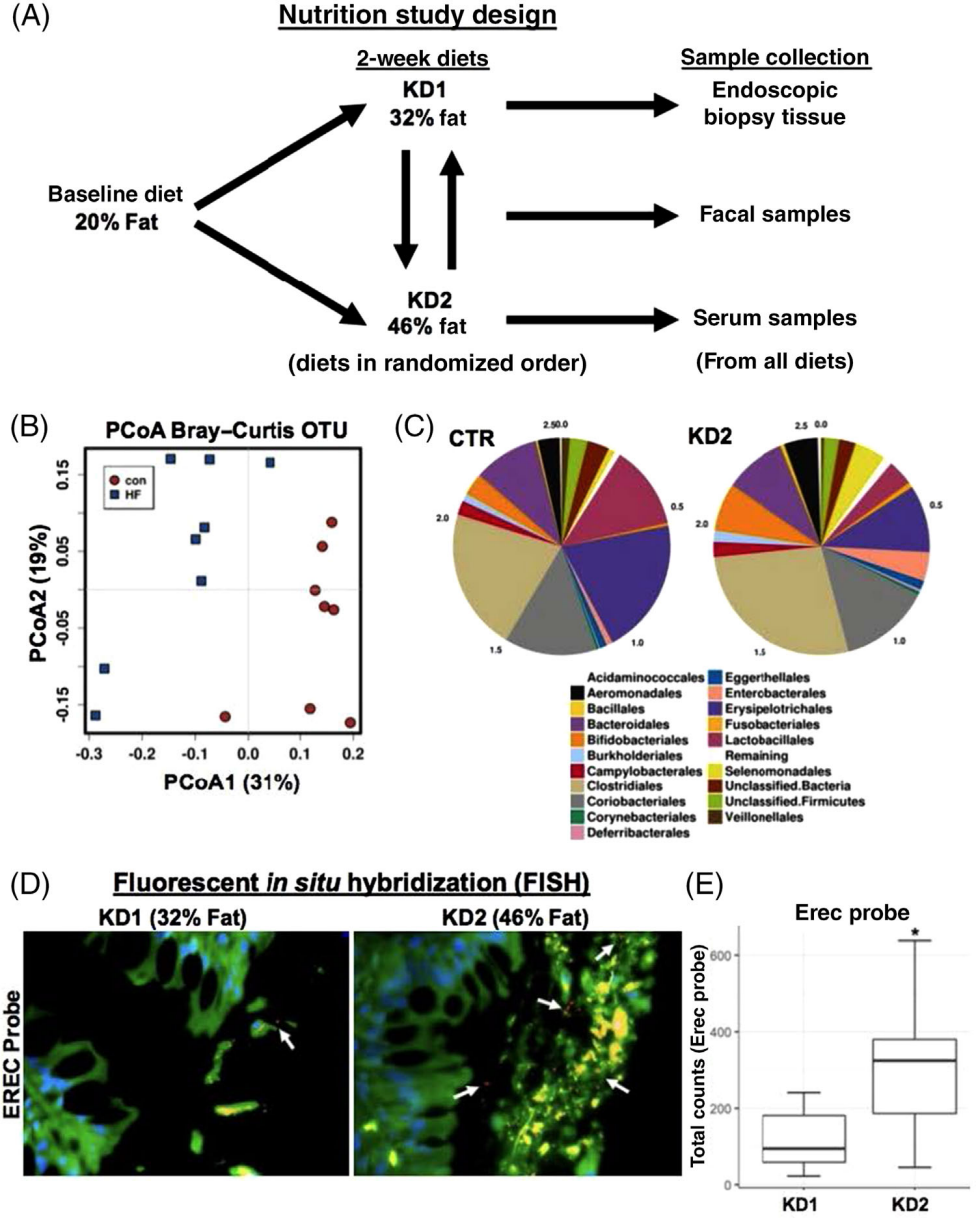

**Figure:**
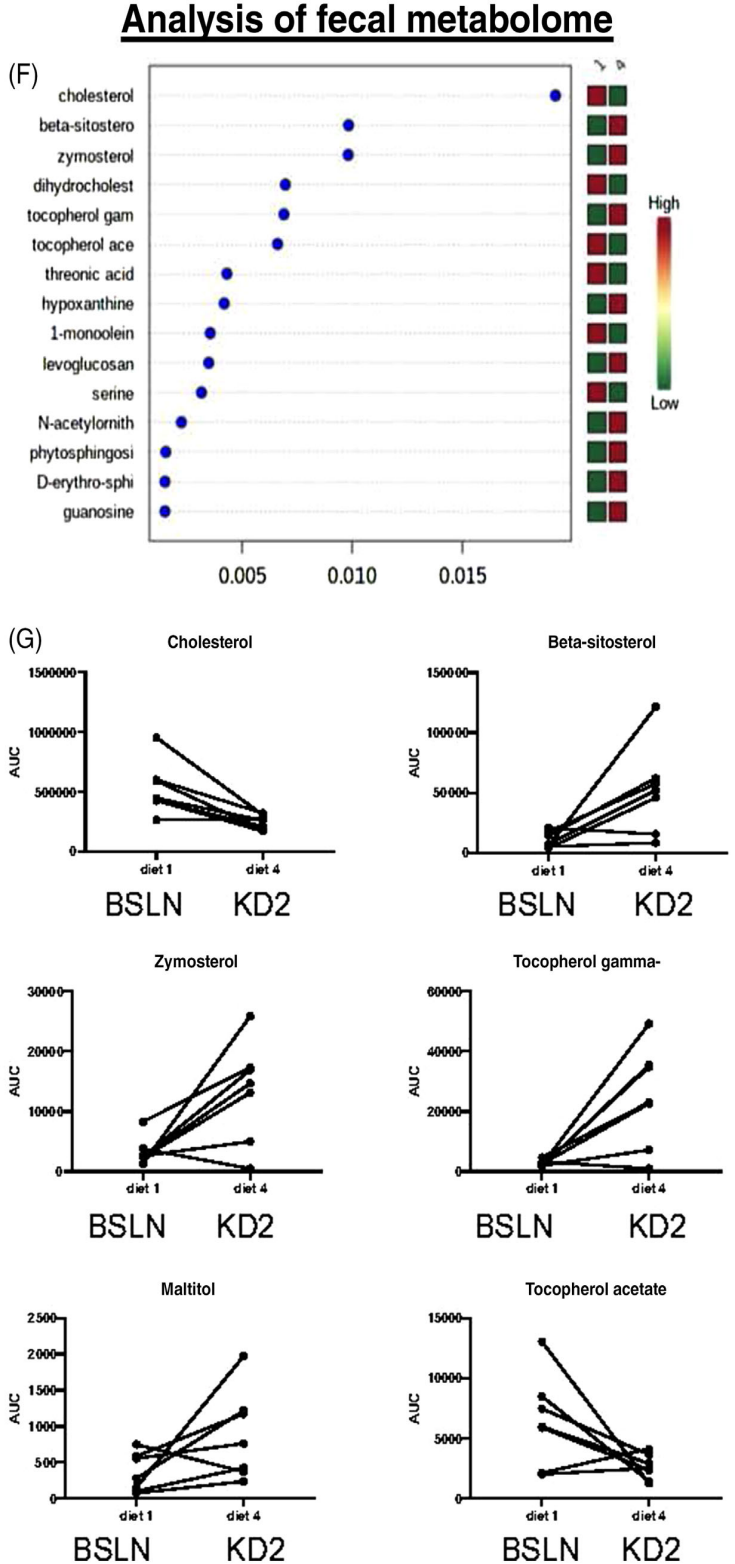

Our first aim was to evaluate the fecal microbiome in dogs after KDs as microbiome changes are thought to be a major mechanism of action for the metabolic effects induced by KDs (full sequencing raw data available in Data S1).^4^ Shallow shotgun sequencing of fecal samples showed distinct clustering and separation between BSLN diet and KD2 by principal coordinate analysis (Figure 1B). Specifically, the abundance of *Lactobacillales* and *Erysipelotrichales* decreased, whereas *Fusobacteriales*, *Bifidobacteriales*, *Selenomonadales*, *Aeromonadales* and *Enterobacteriales* increased with KD2 versus BSLN diet (*p* < .05) (Figure 1C), indicating diet‐specific effects similar to those previously observed in humans on KD. Fecal microbiota changes may not always represent the composition of the microbiome in the upper GI tract. Next, we therefore investigated mucosal‐associated bacteria using fluorescent in situ hybridization (FISH) on intestinal biopsies. The number of mucosal *Clostridia* spp. (Erec probe) was significantly increased (>2‐fold) after KD2 versus KD1 feeding in the colon (*p* = .02) (Figure 1D,E), an effect also previously recognized in humans.

Next, we investigated whether the intestinal dysbiosis was associated with a change in the function of the fecal microbial species using targeted metabolomics. In a random forest analysis, which identified the metabolites with the highest degree of separation between diets, cholesterol was the most significantly altered fecal metabolite and was decreased after the switch to KD (Figures 1F and 2A,B). Overall, most significant changes were related to cholesterol/steroid metabolism as well as the primary bile acid synthesis pathways (Figures 1G and 2C–F, raw data in Data S2).

**FIGURE 2:**
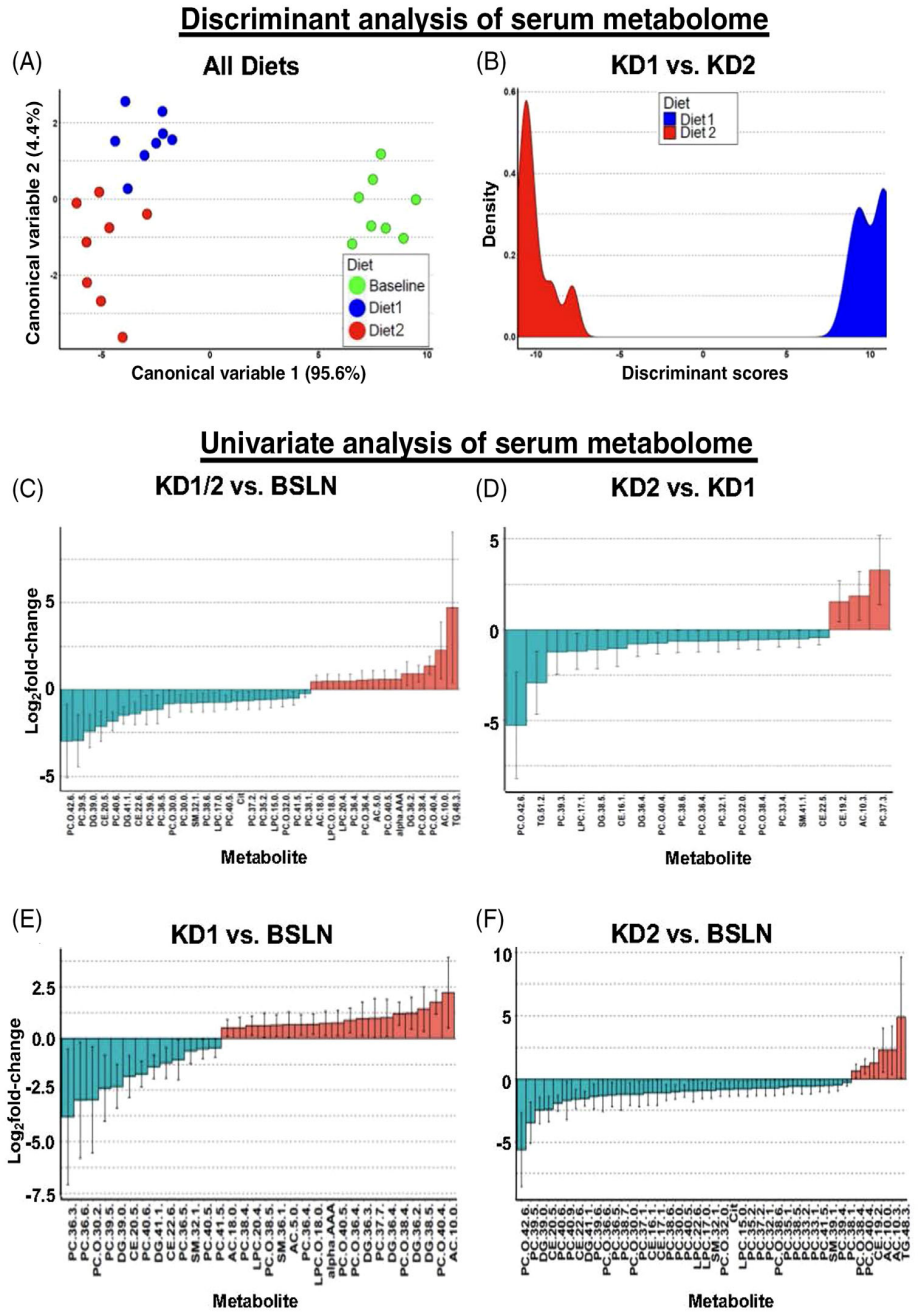
Ketogenic diet (KD) rapidly alters the canine serum metabolomic profile. Discriminant analysis graphs of serum metabolites significantly changed in eight healthy dogs: (A) between all three diets, baseline (BSLN: 28.9 g/1000‐kcal ME; KD1: 64.1 g/1000‐kcal ME; KD2: 79.3 g/1000‐kcal ME) or (B) between KD1 and KD2. Further details of canonical variables and discriminant analysis are shown in Tables S2A and S2B. (C–F) Univariate analysis of individual serum metabolites in healthy dogs after 2‐week diets. Waterfall plots of serum metabolites significantly (*p* < .05) changed in eight healthy dogs for (C) both KD1 and KD2 versus BSLN, (D) KD2 versus KD1, (E) KD11 versus BSLN, or (F) KD2 versus BSLN. Error bars for graphs in (C–F) represent 95% confidence intervals. AC, acylcarnitine; alpha‐AAA, alpha‐aminoadipic acid; CE, cholesteryl ester; Cit, citrulline; DG, diglyceride; LPC, lysophosphatidylcholine; PC, phosphatidylcholine; SM, sphingomyelin; TG, triglyceride.

Changes in mucosal bacteria and fecal microbiome were accompanied by altered serum metabolome analysed by targeted mass spectrometry (Data S3). Discriminant analysis revealed a clear clustering and separation between all three diets (Figure 2A,B), and univariate statistical analysis showed that the most significantly (*p* < .05) changed metabolites were serum lipids (Figure 2C–F). These effects of KD could therefore be interpreted as beneficial to counteract tumor stem cells that are reliant on glycophospholipids.^5^

Many tumor types are more dependent on conditionally essential amino acids, and, especially, branched‐chain amino acids, in order to keep up with the metabolic demands of their highly proliferative growth.^6^ KDs in this study induced significant decreases in the AA valine and methionine, both of which have previously been implicated as conditionally essential AA in tumours.^6^ In addition, the serum concentration of serotonin was increased in our dogs after KDs. High serotonin levels with a concomitant decrease in kynurenine could play a role in anti‐tumor effects as well, as kynurenines have been found to increase tumor proliferation through their growth‐enhancing effects. Collectively, the serum metabolome changes seen in this study indicate the potential for the dietary interventions to affect essential lipid and protein metabolism in cancer stem cells,^7^ which could translate into significant anti‐tumor effects in cancer‐bearing dogs.

Next, we explored whether short‐term feeding of KD modulates intestinal stem cell numbers and proliferation indices. Endoscopic biopsies collected from the dogs fed KD2 revealed that *LGR5+* and Ki‐67+ stem cells in the colon were significantly increased compared to both BSLN and KD1 feeding, respectively, as determined by RNA in situ hybridization (RNA‐ISH) and immunohistochemistry (IHC), respectively (Figure 3A–F). These results indicate a shift towards a less differentiated and more proliferative colonic epithelial phenotype, and were replicated in the ileum (Figure 3G–L; Table S3, Data S4). Whether these changes are indicative of tumorigenic changes (e.g., by promoting mutagenesis in colon and hepatic cancer stem cells,^8^ ^)^ or anti‐proliferative changes (by mimicking metabolic effects of starvation that decrease tumor growth overall), needs further investigation.

FIGURE 3(A–F) Markers of intestinal stem cells and proliferation are increased in colon and mucosal tissue after short‐term ketogenic diet (KD). Increased mucosal staining of LGR5+ stem cells marker and Ki‐67 proliferative marker after KD2 in the colon of eight healthy Beagle dogs. Representative photomicrographs of colon and ileal biopsies from the same dog before (BSLN: 28.9 g/1000‐kcal ME) and after 2‐week exposure to KD2 (79.3 g/1000‐kcal ME), with (A–C) LGR5+ gene expression (in red) visualized by RNA‐*ISH* (RNAscope) using canine specific oligonucleotide probe for LGR5 mRNA (A–C), and Ki‐67 protein levels (in brown) analysed by IHC (D–F). Nuclei counterstained with haematoxylin (blue). Bar graphs of ImageJ analysis of total area of (C) LGR5+ or (F) Ki‐67 staining. Error bars indicate mean ± SEM. *p* < .05. Scale bar = 100 μm (A and B) or 200 μm (D and E). (G–L) Increased mucosal staining of LGR5+ stem cell marker and Ki‐67 proliferative marker after HFD in ileum tissue of healthy dogs. Ileum tissue biopsies from dogs fed BSLN (28.9 g/1000‐kcal ME) versus KD2 (79.3 g/1000‐kcal ME) were stained for LGR5+ (RNA‐ISH) and Ki‐67+ (IHC). LGR5+ expression (red) by RNA‐ISH (G and H) or for Ki‐67 protein (brown) by IHC (J–L), as shown in representative pictures at 40× magnification from the same dog. Graphs of ImageJ analysis of total area of LGR5+ or Ki‐67 staining, normalized to total area of haematoxylin (blue) staining. Error bars indicate mean ± SEM. *p* < .05. Scale bar = 100 μm.

**Figure:**
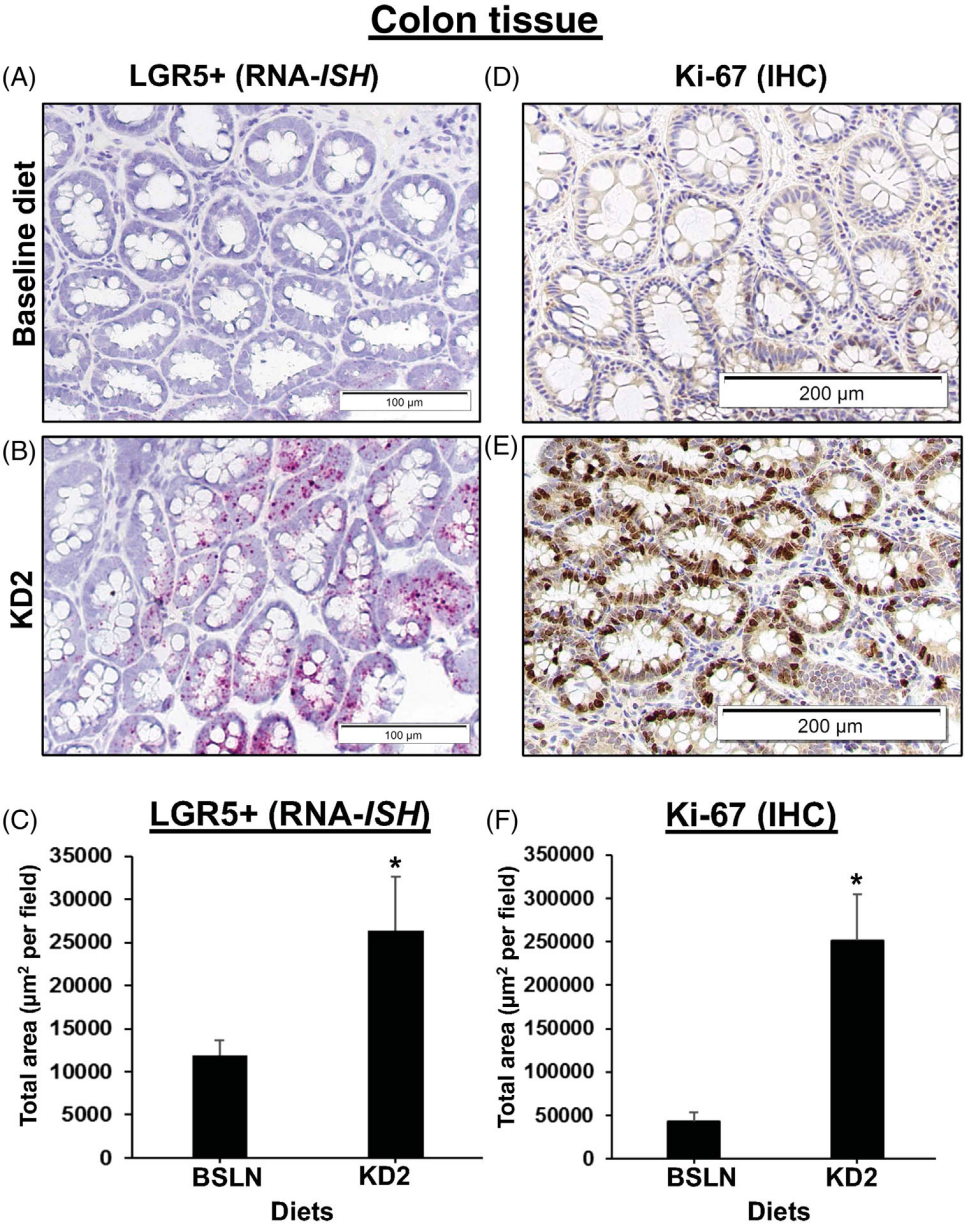

**Figure:**
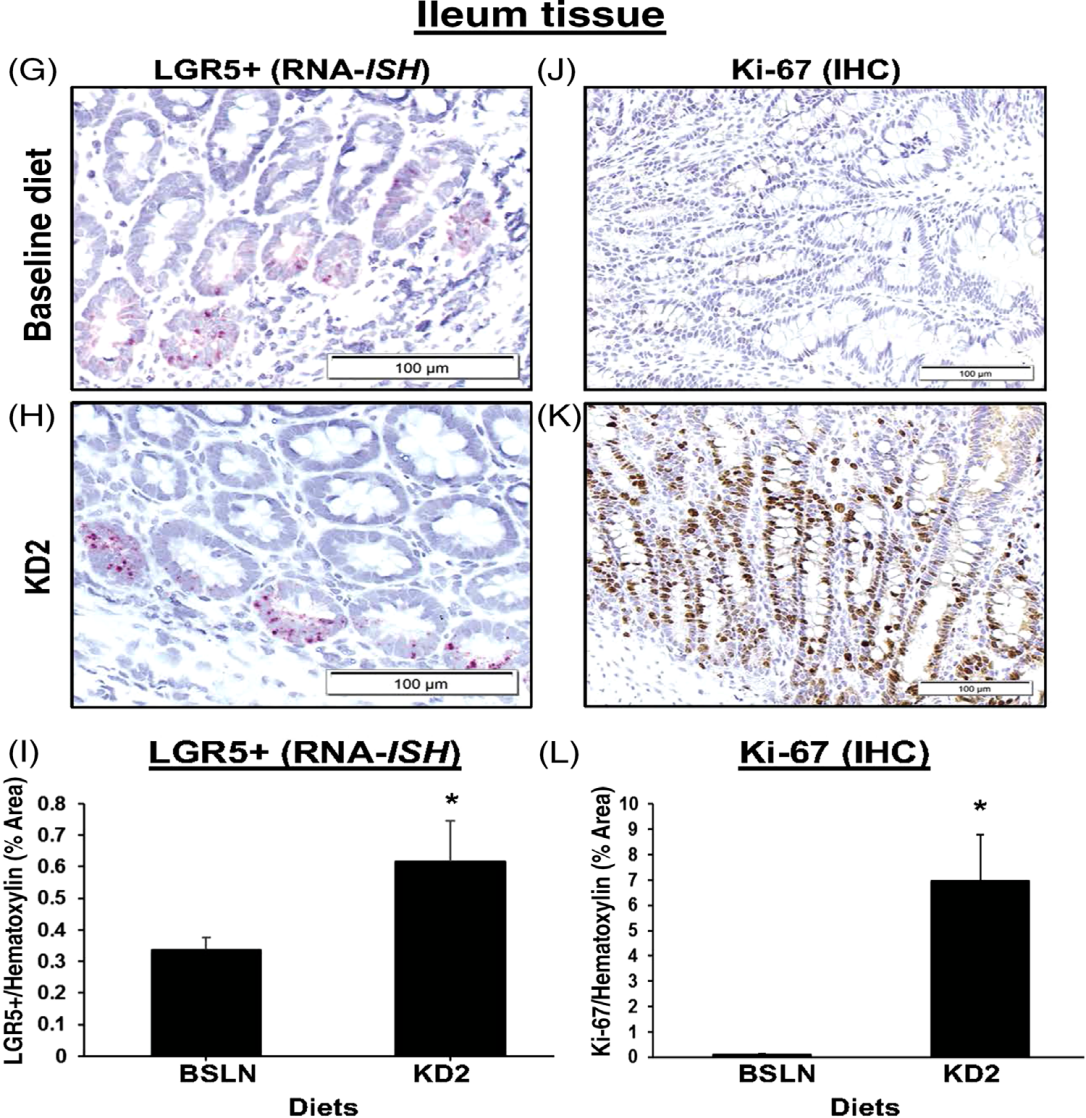

To mechanistically investigate the effect of individual fatty acids in canine intestinal epithelia in vitro, we used palmitic acid (PA) as a proxy for KD‐induced in vivo responses in canine enteroids.^9^ The incubation of healthy canine ileal enteroids with PA mimicked the stem‐cell enhancing, proliferative effects of KD that had been seen in vivo in a dose‐dependent manner (Figure S1). This response was accompanied by an increased expression of the nuclear receptor transcription factor peroxisome proliferator‐activated receptor‐γ (*p* < .05) (Figure S2), similarly to what has previously been described in mouse enteroids stimulated with PA.^10^

In summary, short‐term feeding of KD in dogs significantly shifted the metabolome towards an anti‐tumorigenic molecular signature, which suggests that dogs diagnosed with clinical analogues of human cancers could prove useful in translational clinical trials investigating the therapeutic efficacy of KD in various tumours (Figure S3).

## CONFLICT OF INTEREST

Dr. Allenspach, Jergens and Mochel declare a COI and are co‐founders of 3D Health Solutions, Inc., Ames, IA, a company aiming at commercializing diagnostic assays based on the culture of canine organoids.

## Supporting information

Figure S1 info

Figure S2 Info

Figure S3 info

Table S1A Dry matter/weight measurement of baseline diet (BSLN), ketogenic diet 1 (KD1; 32.1% fat) and ketogenic diet 2 (KD2; 46.5% fat)

Table S2B Discriminant analysis between all three diets (BSLN, KD1 and KD2) shown in Figure 2A.
Table S3 RNAseq analysis of changes in expression of select genes after KD2 versus BSLN in ileum tissue

Supporting Information

## Data Availability

All raw data used in the analyses are available either in the main text or in the Supporting Information or hyperlinks attached to the manuscript (Data S1–Data S4).
