# Supplementary figures and images for: Ketogenic diets in healthy dogs induce gut and serum metabolome changes suggestive of anti‐tumourigenic effects: A model for human ketotherapy trials
Source: Clin Transl Med. 2022 Sep 23;12(9):e1047. doi: 10.1002/ctm2.1047 (PMC9506423; doi:10.1002/ctm2.1047)

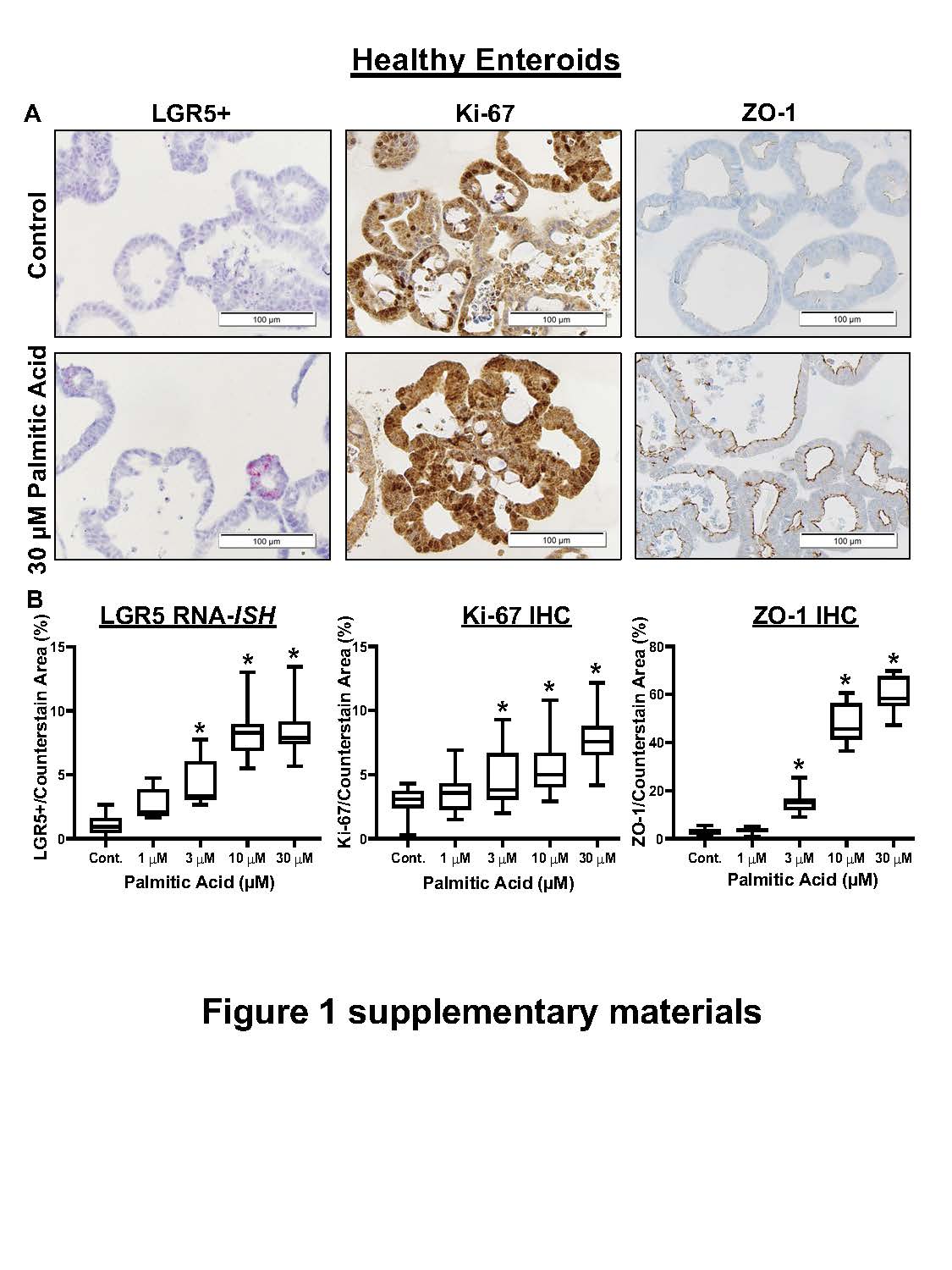

Supplement: Supplementary file 1 — Figure S1 info [file CTM2-12-e1047-s006.jpg]

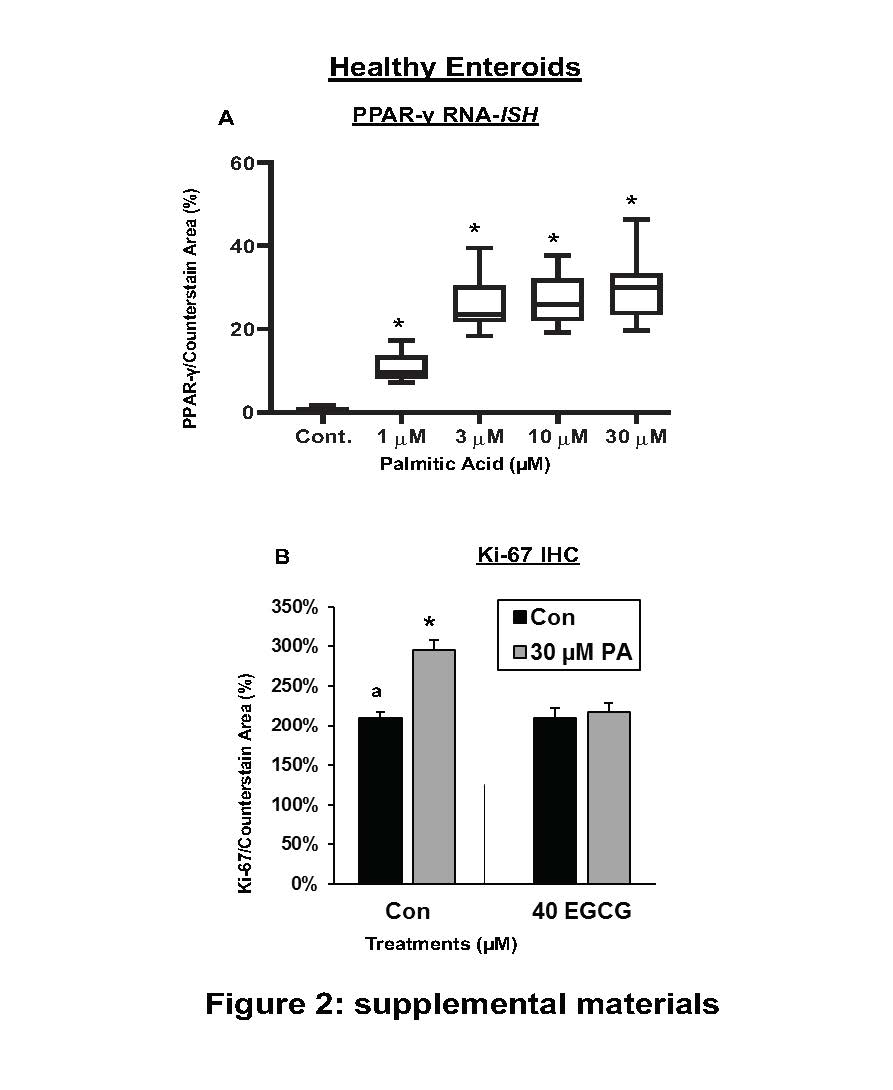

Supplement: Supplementary file 2 — Figure S2 Info [file CTM2-12-e1047-s003.jpg]
