# Supplementary material for: Ketogenic diets in healthy dogs induce gut and serum metabolome changes suggestive of anti‐tumourigenic effects: A model for human ketotherapy trials
Source: Clin Transl Med. 2022 Sep 23;12(9):e1047. doi: 10.1002/ctm2.1047 (PMC9506423; doi:10.1002/ctm2.1047)
Supplement: Supplementary file 3 — Figure S3 info [file CTM2-12-e1047-s005.docx]

**Appendix: Supplementary Materials for Letter to Editor**

**Table S1A.** Dry matter/weight measurement of Baseline diet (BSLN), ketogenic diet 1 (KD1; 32.1% fat) and ketogenic diet 2 (KD2; 46.5% fat).

| Item | 0%= BSLN | Plus 2% canola oil=KD1 | Plus 6% canola oil=KD2 |  |
| --- | --- | --- | --- | --- |
|  |  |  |  |  |
| DM, % | 22.15 | 24.85 | 26.74 |  |
| Moisture, % | 77.85 | 75.15 | 73.26 |  |
| OM, % | 88.96 | 90.74 | 91.6 |  |
| Ash, % | 11.05 | 9.27 | 8.41 |  |
| CP, % | 46.88 | 42.72 | 38.19 |  |
| CP, % by kcal | 30.90 | 26.86 | 22.78 |  |
| Fat, % | 32.05 | 37.15 | 46.49 |  |
| Fat, % by kcal | 47.54 | 52.56 | 62.40 |  |
| Total Dietary Fiber, % | 3.41 | 3.34 | 3.2 |  |
| Total Dietary Fiber, % by kcal | 2.25 | 2.10 | 1.91 |  |
| Total Starch, % | 1.08 | 1.06 | 1.02 |  |
| Total Starch, % by kcal | 0.71 | 0.67 | 0.61 |  |
| GE, kcal/kg | 6068.01 | 6361.67 | 6705.12 |  |
| ME, kcal/kg | 4596.4 | 4916.15 | 5418.15 |  |

Definitions: DM, dry matter; OM, organic matter; CP, crude protein; NFE, nitrogen free extract (soluble carbohydrate, i.e. sugar and starch); GE, gross energy; ME, metabolizable energy. Analysis was performed at the Iowa state Comparative Nutrition Laboratory, Ames, IA.

**Table S1B:** Ingredient composition of the baseline (BSLN) diet

| Diet Ingredients | |
| --- | --- |
| Baseline | Chicken, chicken broth, chicken liver, carrots, peas, dried egg product, guar gum, carrageenan, ground flaxseed, potassium chloride, salt, cassia gum, minerals (zinc amino acid chelate, iron amino acid chelate, copper amino acid chelate, manganese amino acid chelate, sodium selenite, potassium iodine), vitamins (vitamin E supplement, thiamine mononitrate, niacin supplement, d-calcium pantothenate, vitamin A supplement, riboflavin supplement, biotin, vitamin B12 supplement, pyridoxine hydrochloride, vitamin D3 supplement, folic acid), choline chloride |

**Table S2A.** Discriminant Analysis between KD1 (32.1% fat) and KD2 (46.5% fat), shown in **Fig. 2B**.

| Metabolite | SCC |
| --- | --- |
| CE.19.2. | -12.01 |
| DG.34.1. | -3.47 |
| LPC.14.0. | -1.82 |
| PC.O.32.0. | 2.56 |
| PC.O.36.5. | 7.27 |
| Taurine | 4.04 |
|  |  |
| *P*-value | <0.0001 |
| *R^2^* | 99.13% |

Definitions: SCC, Standardized Canonical Coefficients; CE, cholesteryl ester; DG, diglyceride; LPC, lysophosphatidylcholine; PC, phosphatidylcholine; *R^2^*, Squared Canonical Correlation.

**Table S2B.** Discriminant Analysis between all three diets (BSLN, KD1, and KD2),

shown in **Fig. 2A**.

|  | SCC | |
| --- | --- | --- |
| Metabolite | CAN1 | CAN2 |
| PC.40.6. | 2.05 | -2.16 |
| LPC.O.18.0. | -1.13 | 1.31 |
| LPC.20.1. | -1.3 | -1.5 |
| AC.18.0. | -1.77 | -0.33 |
| PC.40.5. | 3.22 | 2.43 |
|  |  |  |
| *P*-value | <0.0001 | 0.0011 |
| *R^2^* | 97.29% | 92.10% |
| [Eigenvalue]^1^ | 95.63% | 4.37% |

Definitions: SCC, Standardized Canonical Coefficients; CAN, Canonical Variable; PC, phosphatidylcholine; LPC, lysophosphatidylcholine; AC, acylcarnitine; *R^2^*, Squared Canonical Correlation.

^1^Percentage of the whole variation accounted for by each CAN

**Table S3.** RNAseq analysis of changes in expression of select genes after KD2 vs. BSLN in ileum tissue.

| **Gene** | **Gene Name** | **Ensembl ID** | **log2FC** | **P-value** | **FDR** |
| --- | --- | --- | --- | --- | --- |
| CXCL8 | Interleukin-8 | ENSCAFG00000003029 | -1.2 | 0.01416 | 0.99941 |
| S100A12 | S100 calcium-binding protein A12 | ENSCAFG00000023324 | -1.2 | 0.03646 | NA |
| RSPO3 | R-spondin3 | ENSCAFG00000001067 | 1.0 | 0.04785 | NA |
| PDK4 | Pyruvate dehydrogenase lipoamide kinase isozyme 4 | ENSCAFG00000002129 | 1.1 | 0.01787 | 0.99941 |
| RSPO2 | R-spondin2 | ENSCAFG00000000696 | 1.2 | 0.02764 | NA |
| PAX5 | B-cell-specific activator protein/paired-box containing 5 | ENSCAFG00000002357 | 3.8 | 0.04247 | 0.99941 |

**Data S1.**

link to NCBI for raw data of sequences of shallow metagenomic sequencing of fecal samples: BioProject accession number PRJNA785003.

**Data S2. (separate file)**

Raw data of fecal metabolome analysis.

**Data S3. (separate file)**

Raw data of serum metabolome analysis.

**Data S4. (separate file)**

Raw data of RNAseq of mucosal tissue samples.
